# Antibacterial and UV Protection Properties of Modified Cotton Fabric Using a Curcumin/TiO_2_ Nanocomposite for Medical Textile Applications

**DOI:** 10.3390/polym13224027

**Published:** 2021-11-21

**Authors:** M. M. Abd El-Hady, A. Farouk, S. El-Sayed Saeed, S. Zaghloul

**Affiliations:** 1 National Research Centre, Institute of Textile Research and Technology, 33 El-Behoth Street, Dokki, P.O. Box 12622, Giza 11311, Egypt; m.aish@qu.edu.sa or asmaa.saleh2015@yahoo.com (A.F.); drsaad_nrc2010@yahoo.com (S.Z.); 2 Department of Physics, College of Science and Arts in Al-Asyah, Qassim University, Buraidah 51452, Saudi Arabia; 3 Department of Chemistry, Faculty of Science, King Khalid University, P.O. Box 9004, Abha 62217, Saudi Arabia; 4 Department of Chemistry, College of Science, Qassim University, Buraidah 51452, Saudi Arabia

**Keywords:** cotton fabric, TiO_2_ nanoparticles, citric acid, Quat 188, curcumin, antibacterial, UV blocking, durability

## Abstract

Medical textiles are one of the most rapidly growing parts of the technical textiles sector in the textile industry. This work aims to investigate the medical applications of a curcumin/TiO_2_ nanocomposite fabricated on the surface of cotton fabric. The cotton fabric was pretreated with three crosslinking agents, namely citric acid, 3-Chloro-2-hydroxypropyl trimethyl ammonium chloride (Quat 188) and 3-glycidyloxypropyltrimethoxysilane (GPTMS), by applying the nanocomposite to the modified cotton fabric using the pad-dry-cure method. The chemistry and morphology of the modified fabrics were examined by Fourier transform infrared spectroscopy, energy-dispersive X-ray spectroscopy, and scanning electron microscopy. In addition, the chemical mechanism for the nanocomposite-modified fabric was reported. UV protection (UPF) and antibacterial properties against Gram-positive *S. aureus* and Gram-negative *E. coli* bacterial strains were investigated. The durability of the fabrics to 20 washing cycles was also examined. Results demonstrated that the nanocomposite-modified cotton fabric exhibited superior antibacterial activity against Gram-negative bacteria than Gram-positive bacteria and excellent UV protection properties. Moreover, a good durability was obtained, which was possibly due to the effect of the crosslinker used. Among the three pre-modifications of the cotton fabric, Quat 188 modified fabric revealed the highest antibacterial activity compared with citric acid or GPTMS modified fabrics. This outcome suggested that the curcumin/TiO_2_ nanocomposite Quat 188-modified cotton fabric could be used as a biomedical textile due to its antibacterial properties.

## 1. Introduction

Nowadays, one of the most promising fields of new textile materials is the manufacturing of antimicrobial-acting medical textiles. In fact, extensive work is being put into improving substances and procedures that could provide safe and adequate protection against various microorganisms. For example, chemical materials such as phenols, nitro compounds, and formaldehyde derivatives, have been extensively used in the manufacturing of antibacterial medical textiles [1,2,3,4]. However, most of these compounds have serious drawbacks in terms of toxicity and poor biodegradability, which make their use quite limited. In order to avoid these issues, the textile industry has been utilizing natural, nontoxic active substances that have no side effects on people or the environment [5,6,7,8].

Fabric modification with nanomaterials designed for enhancing textile properties, such as antibacterial properties [7,9], UV protection [10] wound healing, self-cleaning and military application [11,12], is widely used. In this process, nanoparticles may be incorporated into fabrics for medical applications without affecting their textile properties. In particular, incorporation of antimicrobial agents in the form of nanoparticles can exhibit high levels of antimicrobial activity as well as excellent durability (both in usage and by repetitive laundering cycles), which is much more superior to metal salts or adsorbed quaternary ammonium compounds that operate by leaching from the treated fabrics and are often reduced by laundering [13,14].

Curcumin is a natural material that is used in medicinal textiles. It is a polyphenolic compound and a yellow pigment derived from the ground rhizomes of the Curcuma longa Linn plant, which has a wide variety of beneficial properties. It has a wide range of pharmacological properties, including anti-inflammatory, antioxidative and anti-cancer properties [15,16]. Curcumin contains two phenolic hydroxyl groups and two carbonyl groups in the center, which can form keto-enol tautomers in solution. When it comes to curcumin modifications, the phenolic group is the most important functional group. It is capable of a wide range of reactions, including nucleophilic substitution with organic acids, epoxide and their derivatives [17]. Several experimental studies have indicated that these two groups exist primarily in the enolic form at room temperature [18]. Unfortunately, pure curcumin has a low solubility, which limits its use in medical and clinical applications [19]. In order to overcome this problem, curcumin is used as a complex with other materials in order to enhance its bioavailability [20,21]. Curcumin’s therapeutic effectiveness is limited because of its low solubility, absorption, metabolism, and bioavailability [22]. In this regard, curcumin research has recently focused on the production of possible delivery systems to improve its aqueous solubility, stability, bioavailability and its controlled delivery at specific sites. In order to achieve this, curcumin is incorporated into titanium dioxide nanoparticles. In addition, for enhanced antibacterial activity, we chose hydrophilic titanium dioxide nanoparticles to conjugate with hydrophobic curcumin. Titanium dioxide nanoparticles are used in a wide range of consumer products, including sunscreens, cosmetics, pharmaceutical additives, and food coloring agents. They are biodegradable [22] and have good biocompatibility with little or no toxicity in vitro and in vivo. As a result, titanium dioxide nanoparticles may be one of the most promising nanoparticles for a broad variety of medical and pharmaceutical applications. Nano titanium dioxide can be used in biomedical and bioengineering applications due to its special properties and high reactivity [23]. Curcumin was recently used to sensitize TiO_2_ for improved photodegradation of dyes [24] and photodegradation of phenols [25]. In addition, a complex of titanium dioxide nanoparticles with curcumin was developed as a wound dressing material using chitosan and polypropylene fabric [26]. The incorporation of positively charged sites, such as cationization, allows for the creation of an electrostatic attraction between the fiber and negatively charged molecules. Cotton cationization yielded new cotton cellulose, which could lead to new uses in cotton pre-treatment and chemical finishing. Previous reports illustrate that cationization of cotton surfaces has been shown to improve silver nanoparticle adsorption [27,28] and dye uptake [29,30].

In the current work, we aimed to develop cellulose-based materials that confer better and more durable antibacterial applications. To achieve this, we prepared a curcumin/TiO_2_ nanocomposite for fabrication on the surface of cotton fabric using the pad-dry-cure method. Titanium dioxide nanoparticles in the composite were used to enhance the stability of the curcumin as well as its fabric finishing capacity. To improve the attraction forces between the nanocomposite and the cotton fabric, we used three different crosslinkers, namely 3-Chloro-2-hydroxypropyl trimethyl ammonium chloride (Quat 188), (3 glycidyloxy) propyltrimethoxysilane (GPTMS) and citric acid. This crosslinking process stabilized the curcumin/TiO_2_ nanocomposite. Finally, the mechanism of action of the modified fabric was reported and its durability and mechanical properties were investigated under different crosslinking schemes.

## 2. Experimental

### 2.1. Materials

Mill bleached pure 100% cotton fabric (138 g/m^2^) was supplied by Misr Company for Spinning and Weaving, Mehalla El-Kobra, Egypt.

### 2.2. Chemicals

3-Chloro-2-hydroxypropyl trimethyl ammonium chloride (69%) of technical grade chemicals (known as Quat 188) was purchased under the commercial name CR-2000 from Aldrich. Titanium dioxide P25 powder was provided by Degussa 3- glycidyloxypropyltrimethoxysilane (GPTMS, 95%) was purchased from ABCR (Karlsruhe, Germany). Curcumin powder (99.8% pure and anhydrous) was purchased from Sigma-Aldrich (Taufkirchen, Germany). Sodium hydroxide, acetic acid, hydrochloric acid and sodium hypophosphite were purchased from Loba Chemie (Mumbai, India). Ethanol was purchased from Fisher chemical (Loughborough, UK)

### 2.3. Preparation of GPTMS Sol

GPTMS sol was prepared by mixing GPTMS (10 mL) with isopropanol water (20/80 mL) and stirred at 25 °C for 20 min, 1.22 mL of 0.01 M hydrochloric acid solution was then added dropwise to the GPTMS solution and stirred for 1 h at room temperature to obtain the silica sol form [31].

### 2.4. Preparation of Curcumin: TiO_2_ Nanocomposites

The solution of 0.5% (*w*/*v*) TiO_2_ nanoparticles was resuspended in 50 mL of isopropyl alcohol. Then, 5% (*w*/*v*) curcumin powder in isopropyl alcohol was prepared with stirring. A measurement of 0.5 mL of this solution was then added dropwise to solution of TiO_2_ with continuous stirring for 3–4 h.

### 2.5. Cationization of Cotton Fabric

Chemical modification of the cotton fabric through cationization was carried out using the pad-dry-cure method. The experimental procedures adopted were as follows. 3-Chloro-2-hydroxypropyl trimethyl ammonium chloride (Quat 188) was mixed with sodium hydroxide solution at a NaOH/Quat 188 M ratio of 2:1. The cotton fabric was padded in 100 mL of the prepared mixture in two dips and two nips and then squeezed to a wet pick up of about 100%. The fabric was dried at 40 °C for 10 min and cured at 120 °C for 3 min. Finally the cotton fabric was washed with cold water and 1% acetic acid, followed by several washing cycles and dried under the normal laboratory conditions.

### 2.6. Coating of Cationized Cotton Fabric with TiO_2_/Curcumin Nanocomposite

Cationized cotton fabrics were padded in 100 mL of the 0.5% (*w*/*v*) solution of the TiO_2_/curcumin nanocomposite prepared solution in two dips and two nips and then squeezed to a wet pick up of 100%. Padded fabrics were dried at 80 °C for 5 min and then cured at 180 °C for 3 min. Treated fabrics were rinsed with hot water, then with cold water, and finally dried at room temperature.

### 2.7. Coating of Cotton Fabric with GPTMS/Curcumin/TiO_2_ Nanocomposite

Solution of 2% (*w*/*v*) GPTMS sol was added to 0.5% (*w*/*v*) solution of the TiO_2_/curcumin nanocomposite with continuous stirring under sonication for 2 h the cotton fabrics were padded in the 100 mL of the previously prepared solution in two dips and two nips and then squeezed to a wet pick up of 100%. Padded fabrics were dried at 80 °C for 5 min and then cured at 180 °C for 3 min. Treated fabrics were rinsed with hot water, then with cold water, and finally dried at room temperature.

### 2.8. Coating of Cotton Fabric with Citric Acid/Curcumin/TiO_2_ Nanocomposite

Aqueous solution of citric acid (30 g/L) with sodium hypophosphite (6% *w*/*w*) was added to the 0.5% (*w*/*v*) solution of TiO_2_/curcumin nanocomposite. The cotton fabrics were padded in 100 mL of the previously prepared solution in two dips and two nips and then squeezed to a wet pick up of 100%. The padded fabrics were dried at 80 °C for 5 min and then cured at 180 °C for 3 min. Treated fabrics were rinsed with hot water, then with cold water, and finally dried at room temperature.

## 3. Characterization

### 3.1. Fourier Transform Infrared Spectroscopy (FT-IR)

FTIR spectroscopy has been extensively used in cellulose research since it presents a relatively easy method of obtaining direct information on chemical changes that occur during various chemical treatments. ATR-FTIR instrument (JASCO, Model IR 4700, Tokyo, Japan) was used to scan from 4000 to 400 cm^−1^ in ATR mode using KBr as supporting material.

### 3.2. Scanning Electron Micrograph SEM/EDX Analysis

SEM/EDX Analysis were carried out by using Tescan scanning electron microscope which contains an energy dispersive X-ray (EDX) spectroscopy system (Model vega3, Brono, Czech Republic).

### 3.3. Antibacterial Test

The antibacterial activity of the treated samples against *Staphylococcus aureus*, (Gram-positive) and *Escherichia coli* (Gram−negative) bacteria were determined using an agar plate. The antibacterial activity of the fabric samples was evaluated by using the disk diffusion method. A mixture of nutrient broth and nutrient agar in 1 L distilled water at pH 7.2, as well as the empty Petri plates, was autoclaved. The agar medium was then cast into the Petri plates and cooled in laminar airflow. Approximately 105 colony-forming units of bacteria were inoculated on the plates and 292 cm^2^ of each fabric sample was planted onto the agar plates. All the plates were incubated at 37 °C for 24 h and examined to ascertain whether a zone of inhibition was produced around the samples.

### 3.4. UV Protection Factor

The ultraviolet protection factor (UPF) was measured using a UV Shimadzu 3101 Spectrophotometer (Shimadzu, Kyoto, Japan). UV Protection and classification according to AS/NZS 4399:1996 were evaluated with a scan range of 200–600 nm.

### 3.5. The Add-On (%) loading

The add-on (%) loading was calculated as follows:

(1) $$Add-on\left(\%\right)=\frac{W_{2}-W_{1}}{W_{1}}\times 100$$

where *W*_1_ and *W*_2_ were the weights of the fabric specimens before and after treatment respectively.

### 3.6. Durability Test

The treated fabric samples were subjected to 20 laundering cycles according the ASTM standard test method (D 737-109 96) to determine the antibacterial durability to washing.

### 3.7. Tensile Strength

The tensile strength of the fabric samples was determined by the ASTM Test Method D-1682-94 (1994). Two specimens for each treated fabric were tested in the warp direction and the average value was recorded to represent the fabric-breaking load (Lb).

### 3.8. Statistical Analysis

Results were expressed as a mean value with its standard deviation (mean ± S.D.) for each sample that was repeated three times (*n* = 3). Statistical analysis was performed using a Student’s *t*-test and the differences were considered as significant at *p*-values below 0.05.

## 4. Results and Discussion

### 4.1. Mechanism of Deposition of the Curcumin/TiO_2_ Nanocomposite on the Surface of Cotton Fabric

**Figure 1:**
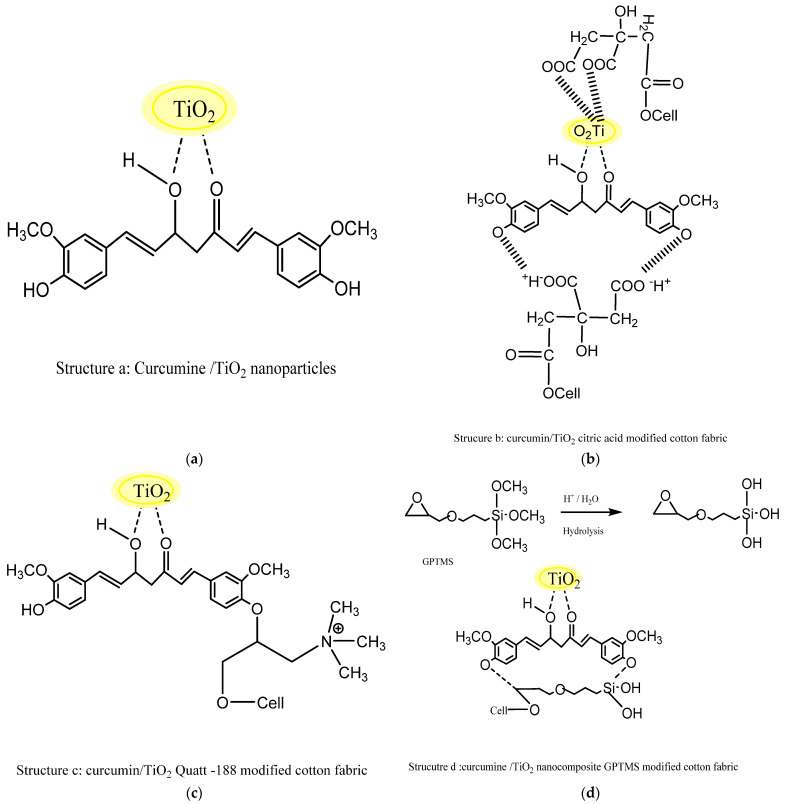
Schematic of the mechanism for the deposition of the curcumin/TiO_2_ nanocomposite on cotton fabrics. (**a**) Structure a: curcumine/TiO_2_ nanoparticles; (**b**) Structure b: curcumin TiO_2_: citric acid modified cotton fabric; (**c**) Structure c: curcumin/TiO_2_, Quatt-188 modified cotton fabric; (**d**) Structure d: curcumine/TiO_2_, nanocomposite GPTMS modified cotton fabric.

Figure 1 illustrates the schematic mechanism of formation and fixation of curcumin/TiO_2_ on the surface of the cotton fabric. At the first stage of this process, formation of the curcumin/TiO_2_ nanocomposite takes place upon addition of the curcumin solution to the TiO_2_ nanoparticle solution, which suggested that the curcumin particles dispersed on the surface of the titanium nanoparticles (Figure 1a). This could be attributed to the high metal chelating potential of the diketone functional group located at the center of the curcumin molecule where the diketone group effectively chelates the TiO_2_ nanoparticles through charge transfer complex formation [24]. Figure 1b illustrates fixation of the curcumin/TiO_2_ nanocomposite on the fabrics modified with citric acid in the presence of sodium hypophosphite, which allows for formation of ester carbonyl linkages [32] with the fiber. After treatment with citric acid modified fabric with curcumin/TiO_2_ nanocomposite, the -OH groups of curcumin in nanocomposite get attached to the functionalized carboxylic group of citric acid on the modified fabric. This resulted in a strong electrostatic interaction of opposite charges between the particles [33]. Interestingly, the negative surface charges induced by the existence of carboxylic acid moieties improved the adsorption affinity of the curcumin/TiO_2_ nanocomposite [34]. In fact, the TiO_2_ nanoparticles exhibited a strong binding to the carboxylic groups of the citric acid-modified fabric through a number of different forms of binding, including weak anion-cation type attractions, hydrogen bonding and coordination-type interaction [35]. The fabrication of the curcumin/TiO_2_ nanocomposite on fabrics modified with Quat 188 is presented in Figure 1c. Clearly, development of an ether linkage between Quat 188 and cellulose resulted from the reaction of Quat 188 with the cotton fabrics [36]. Deposition of curcumin/TiO_2_ nanocomposite on Quat 188-modified fabric was due to strong ionic and van der Waals forces between the –OH groups of the curcumin molecule and the quaternary ammonium modified cotton fabric. Figure 1d illustrates the final treatment of GPTMS-modified cotton fabric using the curcumin/TiO_2_ nanocomposite. GPTMS was pre-hydrolyzed for conversion of the alkyl oxygen groups (-OCH_3_) to hydroxyl groups (-OH). The fabric was modified by GPTMS through ether crosslinking within the cotton fabric via the reaction of epoxy groups of GPTMS with hydroxyl groups of cellulose structure [31]. This allowed for hydrogen bond formation between the GPTMS-modified cotton fabric and the hydroxyl groups of the curcumin molecule [37].

### 4.2. FTIR Analysis

**Figure 2:**
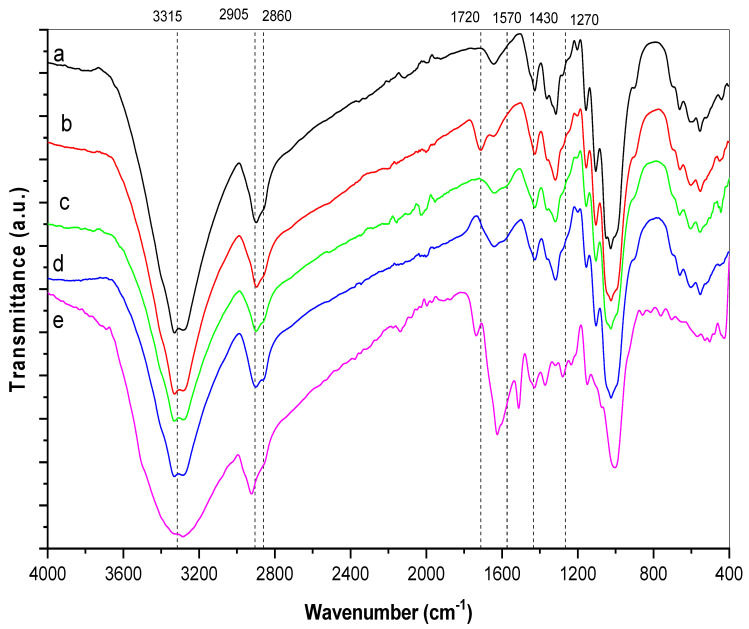
FTIR spectrum of untreated cotton fabric (**a**), curcumin/TiO_2_–citric-modified cotton fabric (**b**), curcumin/TiO_2_– Quat 188-modified cotton fabric (**c**), curcumin/TiO_2_–GPTMS-modified cotton fabric (**d**), and curcumin powder (**e**).

Existence of functional groups on the treated cotton fabric was investigated by fourier transform infrared spectroscopy. Figure 2 illustrates the FTIR spectrum for the untreated cotton fabric (a), curcumin/TiO_2_–citric-modified cotton fabric (b), curcumin/TiO_2_–Quat 188-modified cotton fabric (c), curcumin/TiO_2_–GPTMS-modified cotton fabric (d), and curcumin powder (e). In the untreated cotton fabric, spectrum (a), a band appears in the range of 3200–3500 cm^−1^, which is attributed to O–H stretching. The presence of C–H, O–H, C–O, and C–O–C vibrations caused the characteristic bands in the range of 1500–800 cm^−1^ [38]. On the other hand, the absorption band at 3310 cm^−1^, which corresponded to the stretching vibration of phenolic O–H, was indicated in spectrum (e) for pure curcumin. Moreover, sharp absorption bands appear in the range from 1430 to 1630 cm^−1^. These bands belong to the –OH, C=O, and C=C groups, respectively (enol). Other bands are observed in the region between 1000 cm^−1^ and 1300 cm^−1^. All bands could be ascribed to the configuration of the symmetric and asymmetric C–O–C groups [39]. Interestingly, spectra (b), (c), and (d) look similar to untreated cotton fabric and curcumin patterns with few significant changes. This could be attributed to the partial interaction of the nanocomposite with the modified cotton fabric. Strong bands in the region 400–600 cm^−1^ were, however, noticeable in spectra (b–d), which corresponded to the Ti–O stretching vibration [24]. This confirmed the deposition of curcumin/TiO_2_ nanocomposite on the surface of the modified fabric. Importantly, spectrum (b) showed a well-developed band at 1720 cm^−1^ that could be assigned to a carbonyl group stretching, implying that cellulose was successfully crosslinked with citric acid via the formation of ester carbonyl linkages [40]. It is obvious from spectrum (c) that a new peak emerged at 1570 cm^−1^, which could be attributed to the quaternary ammonium groups. Clearly, spectrum (d) revealed two new bands at 2905 cm^−1^ and 2860 cm^−1^, which could be aligned with the stretching of the methylene groups from the GPTMS molecules.

### 4.3. Surface Morphology of the Cotton Fabrics

**Figure 3:**
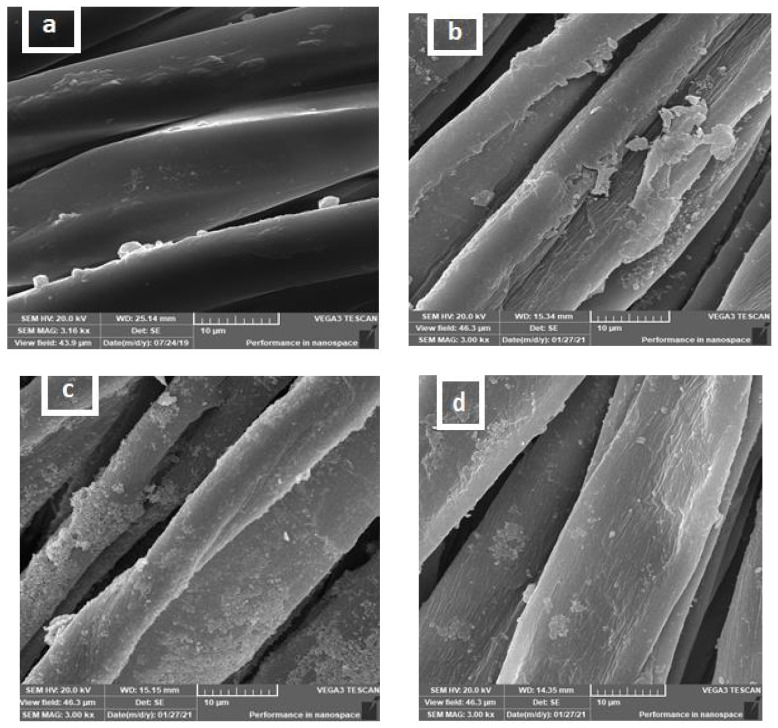
SEM images of control cotton fabric (**a**), curcumin/TiO_2_–citric-modified cotton fabric (**b**), curcumin/TiO_2_–Quat 188-modified cotton fabric (**c**), curcumin/TiO_2_–GPTMS-modified cotton fabric (**d**).

SEM images are used to study the morphology of the fabric surface [41]. Figure 3 displays the variations in unmodified and modified cotton fabric morphology. Figure 3a shows that the unmodified cotton fabric formed a fiber with a smooth surface. Figure 3b–d illustrates the effect of deposition of different modifications of cotton fabric. All modified samples show homogenous distribution of the curcumin/TiO_2_ nanocomposite with less agglomeration in some points. In addition, there were no bridges between cotton adjacent fibers, which is desirable as air and vapor permeability is required for their potential application as wound dressings and medical materials. Figure 3b shows that the curcumin/TiO_2_ nanocomposite with citric acid-modified cotton fabric formed cracked fibers, which could be attributed to the effect of crosslinking with citric acid. On the other hand, Figure 3c shows a higher dense layer in the curcumin/TiO–Quat 188-modified cotton fabric compared with the curcumin/TiO_2_–GPTMS-modified cotton fabric. This may be due to the cationic modification of cellulosic fibers bearing a positive charge, resulting in higher deposition of curcumin/TiO_2_ nanocomposite on their surfaces [42].

### 4.4. EDX Analysis

**Figure 4:**
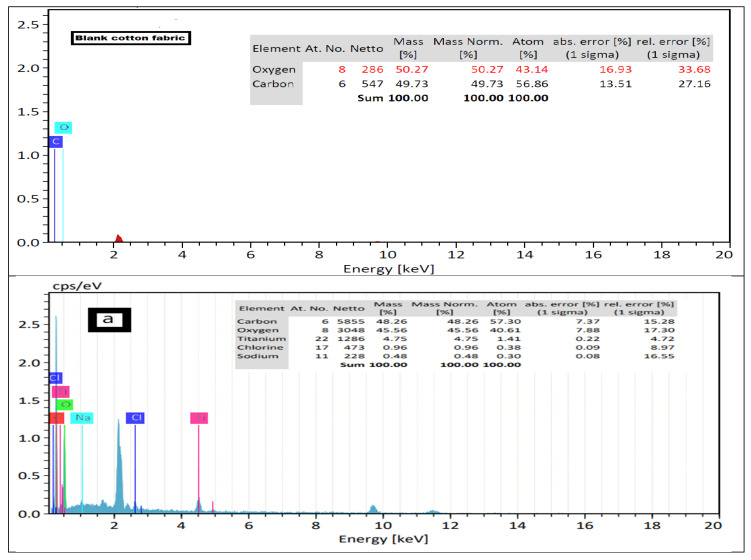

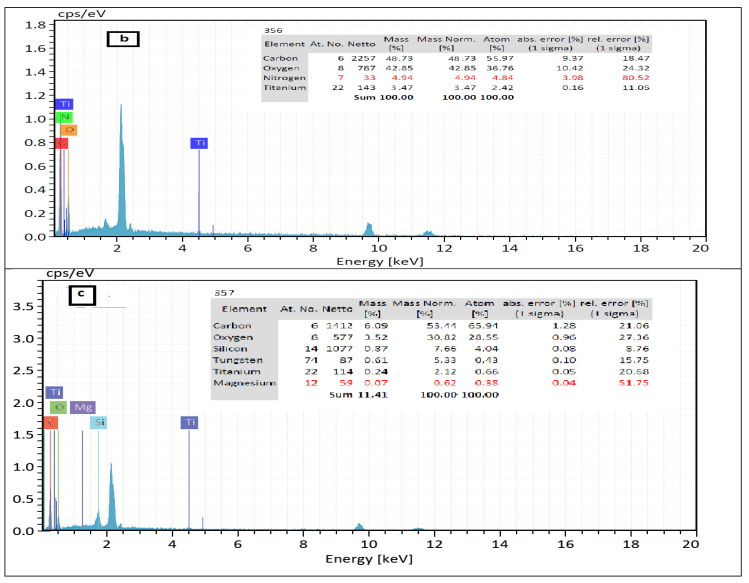
EDX analysis of blank cotton fabric, (**a**) curcumin/TiO_2_–citric-modified cotton fabric, (**b**) curcumin/TiO_2_–Quat 188-modified cotton fabric, and (**c**) curcumin/TiO_2_–GPTMS-modified cotton fabric.

Elemental analysis of the cotton fabric after modification was determined using the EDX spectrum. Figure 4 (blank cotton fabric) shows that the atomic percentage of carbon was 56.86% and oxygen was 43.14%. Interestingly, Figure 4a shows the atomic percentage of carbon as 57.30% and oxygen as 40.61% along with the titanium element as 1.41% for curcumin/TiO_2_–citric-modified cotton fabric. Moreover, Figure 4b shows that the cotton fabric modified by curcumin/TiO_2_–Quat 188 had the atomic percentage of carbon at 55.97, oxygen at 36.76%, titanium at 2.42% and a new peak for nitrogen at 4.84%. Such variation was due to the etherification reaction involved in the cationization process on the cotton fabric surface. On the other hand, the cotton fabric that was modified using curcumin/TiO_2_–GPTMS, Figure 4c, had the atomic percentage of carbon as 65.91%, oxygen as 28.55%, titanium 0.66%, and a new peak for silicon at 4.04%. On the basis of these results, the higher peaks observed for the titanium element in Figure 4b can be related to the higher content of the curcumin/TiO_2_ nanocomposite deposited on the Quat 188-modified cotton fabric.

### 4.5. Antibacterial Activity

The antibacterial activity of curcumin/TiO_2_-modified cotton fabric with various treatments was analyzed against representative microorganisms of open interest, both Gram-positive (*S. aureus*) and Gram-negative (*E. coli*) strains using the agar diffusion method were used. The antibacterial effect for all treatments ranged from 10 mm to 20 mm of clear zone of inhibition. Results mentioned in Table 1 indicated that *Escherichia coli* had a higher response than *Staphylococcus Aureus,* which could be due to variations in bacterial cell wall organization structure. Gram-positive bacteria had a thicker layer cell than Gram-negative bacteria, which served as a barrier to the spread of active ingredient into the cytoplasm, thereby protecting the cell wall [43]. On the other hand, TiO_2_ nanoparticles that coated the modified cotton fabric showed higher antimicrobial activity. This could be attributed to the effect of a metal ion that may cause cytoplasmic leakage, protein denaturation, and enzyme malfunctions. Reactive oxygen species (ROSs) are generated by photoactive metal oxides, which can cause oxidative stress, cell content leakage, and DNA damage [26,44]. Since microbes are inhibited, these ROSs can oxidize lipids and lipopolysaccharides. In addition, the curcumin molecule in the cotton fabric modified with the curcumin/TiO_2_ nanocomposite resulted in higher antibacterial activity. As reported before, curcumin, being a lipophilic molecule, can intercalate into the lipopolysaccharide-containing cell membrane and, thereby, increase the permeability of Gram-negative bacteria. Furthermore, it has been reported that the key mechanism involved in the killing action of curcumin is through the disordering of 1,2-dipalmitoyl- sn-glycero-3-phosphocholine (DPPC) membranes found in both *S. aureus* and *E. coli* [45]. Since curcumin can easily form a complex with titania, it may have been able to break through the bacteria’s cell wall and enter the cell. This could have disrupted cell organelles and induced lysis, that killed bacteria [26]. In addition, cotton fabric modified with Quat 188 showed better antibacterial properties compared with fabric modified by either citric acid or GPTMS. From the above results, hindrance against pathogenic strains was accomplished in the following order: curcumin/TiO_2_ nanocomposite-modified, Quat 188-cationized fabric > curcumin/TiO_2_ nanocomposite-modified, crosslinked fabric with citric acid/SHP > curcumin/TiO_2_ nanocomposite-modified fabric with GPTMS.

Durability to washing cycles is shown in Table 1. Clearly, raising the number of washing cycles to 20 caused a small decrease in the antibacterial properties of the washed treated fabrics. This could be attributed to the effect of the crosslinkers used (citric acid, Quat 188 and GPTMS) [46]. Crosslinkers were used in order to enhance bonding between the curcumin/TiO_2_ nanocomposite and the cellulosic chains of cotton fabric. Thus, favorable washing durability was obtained.

### 4.6. UV Blocking

UPF values were measured to determine the UV-radiation protection characteristics of untreated cotton fabrics and nanocomposite-modified fabrics and the results are shown in Table 2. Textiles can be classified into three protection groups according to BS EN 13758-2:2003 [47]: good (UPF range 20–29), very good (UPF range 30–40), and excellent (UPF range > 40).

The calculated UPF value of untreated cotton fabric is 4.5. The UPF of coated cotton fabric varies from 20 to 55, which is higher than the untreated fabric. In addition, the results in Table 2 indicate that curcumin/TiO_2_-modified cotton fabric increases the UPF values. Inspection of Table 2 revealed that the UPF value of TiO_2_-coated cotton fabric was 20. The increased UPF could be attributed to the semi-conductive properties of the TiO_2_ nanoparticles, which can absorb ultraviolet photons [32]. On the other hand, there was a significant increase in UPF values for TiO_2_-modified cotton fabric. The values varied from 23 to 30 due to the effect othef different treatments according to the following order:

TiO_2_-coated, cationized cotton fabric > TiO_2_-coated, crosslinked fabric with citric acid/SHP > TiO_2_-coated, pretreated cotton fabric with GPTMS.

Moreover, the results in Table 2 indicate that curcumin/TiO_2_-modified cotton fabric increased the UPF values, which varied from 38 to 55 and were graded from very good to excellent protection. This could be a result of the effectivity of the curcumin molecule in increasing the ultraviolet protection of cotton fabric. It can be concluded that the unmodified cotton fabrics exhibited a rather poor UV protection value due to the inability of the cellulose in UV absorption. In contrast, all the nanocomposite-modified fabrics showed better UV blocking properties than the unmodified fabric. The increasing UPF values ofthe curcumin/TiO_2_-modified cotton followed the order:

Curcumin/TiO_2_ nanocomposite-modified, Quat 188-cationized fabric > TiO_2_-coated, crosslinked cotton fabric with citric acid/SHP > curcumin/TiO_2_ nanocomposite-modified cotton fabric with GPTMS.

### 4.7. Add-on and Tensile Strength Measurements

Table 3 shows the percentage of values for add-on measurements and mechanical properties of chemically modified cotton fabric. The amount of chemicals deposited on the cotton fabric during modification was indicated by the add-on values. The results showed that for GPTMS-modified cotton fabric, the add-on values were between 8.45% and 12.57%, whereas the modification of samples with citric acid and cationized agent caused a significant increase in the add-on ranging between 8.65% and 18.87%, and 9.55% and 15.45% respectively.

On the other hand, Table 1 showed significant decreases in values of tensile strength. This may be attributed to the effect of different modifications and crosslinking agents, resulting in damaged cellulose chains.

## 5. Conclusions

In this work, we developed materials based on the fabrication of curcumin/TiO_2_ nanocomposite on the surface of cotton fabric via the pad-dry-cure method. To achieve this goal, cotton fabric was modified with different crosslinkeres, namely citric acid, Quat 188 and GPTMS. Crosslinkers were used to improve the adhesion between cotton fabrics and the prepared curcumin/TiO_2_ nanocomposite. The prepared nanocomposite-modified fabrics were confirmed using FTIR, SEM and EDX. It has been concluded that curcumin/TiO_2_ nanocomposite-modified, Quat 188-cationized fabric showed the highest antibacterial activity compared with either curcumin/TiO_2_ nanocomposite-modified, crosslinked fabric with citric acid/SHP or curcumin/TiO_2_ nanocomposite-modified fabric with GPTMS. Moreover, the curcumin/TiO_2_ nanocomposite-modified, Quat 188-cationized fabric exhibited higher efficiency against Gram-negative bacteria than Gram-positive ones. The durability of curcumin/TiO_2_ nanocomposite-modified cotton fabric showed negligible reduction in antibacterial activity as a result of the crosslinker used. These results are promising for the treatment of fabrics for medical applications. Cationic modification can be used for the modification of cotton fabric to increase curcumin/TiO_2_ nanocomposite adsorption on the surfaces and produce stronger antibacterial activity. The results of UV protection also revealed that curcumin/TiO_2_ nanocomposite-modified, Quat 188-cationized fabric acquired UPF values higher than 50, which indicated excellent UV protection properties.

## Figures and Tables

**Table 1:** Antibacterial activity and durability properties.

Table	Inhibition Zone (mm/1 cm Sample)			
	G− *Escherichia coli*		G+ *Staphylococcus aureus*	
No. of washing cycles	1	20	1	20
Untreated cotton fabric	0	0	0	0
TiO_2_-coated, crosslinked fabric with citric acid/SHP	17	16	15	14
Curcumin/TiO_2_ nanocomposite-modified, crosslinked fabric with citric acid/SHP	18	16	16	14
TiO_2_-coated, Quat 188-cationized fabric	19	17	14	13
Curcumin/TiO_2_ nanocomposite-modified, Quat 188- cationized fabric	22	19	16	14
TiO_2_-coated, pretreated fabric with GPTMS	12	10	11	10
Curcumin/TiO_2_ nanocomposite-modified fabric with GPTMS	14	13	12	10

**Table 2:** UPF values of cotton fabric treated with different conditions.

Treatment	UPF Value	UV-A	UV-B	UV Protection
Untreated cotton fabric	4.5	26	18.8	Non-ratable
TiO_2_-coated cotton fabric	20	15.29	13.69	Good
TiO_2_-coated, crosslinked cotton fabric with citric acid/SHP	27	7.74	6.11	Good
Curcumin/TiO_2_ nanocomposite-modified, crosslinked cotton fabric with citric acid/SHP	50	3.5	3.1	Excellent
TiO_2_-coated, Quat 188-cationized cotton fabric	30	5.24	5.23	Very good
Curcumin/TiO_2_ nanocomposite-modified, Quat 188-cationized fabric	55	2.7	2.5	Excellent
TiO_2_-coated, pretreated cotton fabric with GPTMS	23	14.1	12.2	Good
Curcumin/TiO_2_ nanocomposite-modified cotton fabric with GPTMS	38	6.1	4.5	Very good

**Table 3:** Add-on measurements and tensile strength with standard deviations of treated cotton fabric.

Treatment	Add on (%)	Tensile Strength (Kg f)
Untreated cotton fabric	0	55 ± 1.04
TiO_2_-coated, crosslinked fabric with citric acid/SHP	9.55 ± 0.1	48 ± 1.3
Curcumin/TiO_2_ nanocomposite-modified, crosslinked fabric with citric acid/SHP	15.45 ± 0.06	45 ± 1.8
TiO_2_-coated, Quat 188-cationized fabric	8.65 ± 0.05	47 ± 1.7
Curcumin/TiO_2_ nanocomposite-modified, Quat 188-cationized fabric	18.87 ± 0.12	44 ± 1
TiO_2_-coated, pretreated fabric with GPTMS	8.45 ± 0.1	51 ± 0.5
Curcumin/TiO_2_ nanocomposite-modified fabric with GPTMS	12.57 ± 0.4	47 ± 1.1

## Data Availability

The data presented in this study are available on request from the corresponding author.
